# The introduction of advanced paramedics into primary care in Northern Ireland: a qualitative descriptive study of the experiences of general practitioners

**DOI:** 10.29045/14784726.2021.12.6.3.1

**Published:** 2021-12-01

**Authors:** Damian Muldoon, Chris Seenan

**Affiliations:** Northern Ireland Ambulance Service; Glasgow Caledonian University

**Keywords:** advanced practice, general practitioner, primary care, qualitative descriptive

## Abstract

**Background:**

Primary care is dealing with an ever-increasing workload. The causes are multi-factorial but include a decreasing number of General Practitioners (GPs), combined with increased numbers of patients with multiple co-morbidities and an ageing population. As a result of these pressures, nursing and allied health professionals are now working within a growing number of advanced practice roles delivering community-based care. One such example is paramedics taking up advanced roles within General Practice settings in Northern Ireland. What is not known, however, is what GPs’ experiences are of these developments.

**Aims:**

To examine the experiences of GPs who have introduced an advanced paramedic into their primary care team in Northern Ireland.

**Design:**

A qualitative descriptive design was chosen as the most suitable approach to allow participants to relay their experiences in their own words within the loose confines of a semi-structured interview.

**Methods:**

Semi-structured interviews were conducted with a group of four purposively selected GPs who had direct experience of the phenomena of interest. These interviews were transcribed verbatim, anonymised and then analysed thematically.

**Results:**

The thematic analysis produced three superordinate themes of *alleviating pressure*, *acceptance* and* psychological well-being*. These were underpinned by seven ordinate themes that were supported using verbatim quotes. These were then discussed and contextualised with themes from existing literature.

**Conclusion:**

Generally, there was widespread support from the GPs for the introduction of advanced paramedics into primary care teams. The reasons were multi-factorial but the reduction in GP workload featured prominently. The participants reported benefits in terms of increased resilience and work–life balance. The capacity to provide a clinician with experience of dealing with acute and emergency presentations, in combination with managing routine procedures, was also reported to be of great importance.

## Introduction

Contemporary NHS strategies aim to combat mounting primary care workloads by encouraging the development of multidisciplinary teams made up of advanced practice professionals equipped to deal with the demands of primary care (NHS England, 2014). Booker and Voss (2019) advocated for an increase in the number of paramedics being employed in primary care, maintaining that the skillset of this under-utilised profession would be of great benefit to GPs.

Community-based paramedicine is an established modality of providing primary healthcare in many countries around the world (Martin and O’Meara, 2019). Paramedics within primary care perform many roles previously undertaken by GPs (Brown, 2017). The introduction of paramedics into primary care teams has been suggested as having direct benefits to GPs (Booker and Voss, 2019; Martin and O’Meara, 2019; Pillin, 2015). However, to date, only one study has explored this development in practice (Schofield et al., 2020) and as this study explored the role from a conceptual standpoint, GPs’ views and experiences of paramedics working in primary care have not been explored.

The first community paramedic programme was introduced in Northern Ireland in 2017. This was a three-year EU-funded programme delivered by ‘Co-operation and Working Together’ (CAWT) and the Northern Ireland Ambulance Service (NIAS). The paramedics involved in the pilot also undertook a Master’s in Advanced Paramedic Practice which ran concurrent with the three years of their primary care placement. Attainment of this award allowed the paramedics to use the title of Advanced Paramedic. The paramedics’ scope of practice in the primary care setting grew as they learned new skills from in-house training combined with the specialist training modules of the university degree. The role undertaken in the practice included telephone triage, clinic-based consultations and home visits. Skills such as blood collection, urine analysis, point-of-care CRP testing, INR testing, simple suturing and wound management as well as advanced assessment of body systems were learned and developed throughout the placement. The commencement of this initiative provided an opportunity to explore the experience of GPs regarding these developments in practice. Therefore, the aim of this study is to explore the lived experiences and views of GPs of working with an advanced paramedic in their primary care team.

## Methods

A qualitative descriptive approach was selected to allow meaning to be acquired from detailed first-person accounts of experiences (Sandelowski, 2000; Smith, 1996).

### Participants and setting

The study aimed to explore the experience of GPs working with advanced paramedics in primary care. Purposive sampling was employed of four of the five GPs who worked in the practice involved in the pilot programme. Homogeneity within the grouping was aimed for to ensure richness and depth of data (Ryan et al., 2007; Sandelowski, 2000). The general participant characteristics are detailed in Table 1.

**Table 1.** Participant demographics.

Participant	Years qualified as a GP	Sessions per week
**Dr A (Locum)**	12	6
**Dr B (Partner)**	22	4
**Dr C (Partner)**	12	9
**Dr D (Partner)**	30	8

Initial gatekeeper approval was sought and received from the practice manager to contact the selected participants. All participants received the study information sheet by email and were allowed at least 24 hours to decide whether to participate. All participants provided written informed consent (see Supplementary 1).

### Data collection

Semi-structured person-to-person audio-recorded interviews were conducted between January and February 2020. These interviews lasted between 30 and 50 minutes and followed the interview guide (Supplementary 2). Each interview was conducted at a mutually convenient place and time. A second paramedic researcher was present at the interviews to provide technical assistance. Participants were afforded the opportunity to listen back to the recordings. These recordings were then transcribed verbatim and anonymised by the author before the initial thematic analysis.

### Data analysis

The study utilised a thematic analysis approach to examine the data, to allow easier identification and subsequent pattern reporting of themes (Braun and Clarke, 2006)

The method of coding which made up the general framework involved a line-by-line examination of the individual transcripts (Larkin et al., 2006), followed by a re-reading of the transcripts to become immersed in the data (Biggerstaff and Thompson, 2008; Sandelowski, 2010). This reflective process had three elements, which involved annotating the descriptive, the linguistic and the conceptual comments (Smith et al., 2009). Developing emergent themes from the dataset of annotations and original text enabled the discovery of patterns and connections. The emergent themes were then organised and synthesised into groups of ordinate themes which formed the development of superordinate themes (Braun and Clarke, 2006). An exercise followed looking for patterns across each case for similar themes and superordinate themes (DeSantis and Ugarriza, 2000), reflecting that the process of analysis is iterative and inductive (Smith, 2007).

The thematic analysis was reviewed independently by a separate researcher (KS) and the results compared, primarily to reduce researcher bias and add rigour to the study, increasing the credibility and dependability of the results (Koch, 2006). The idiographic approach of the study is demonstrated by using verbatim quotations in the results section, which permits the retrieval of a particular statement attributed to a specific participant.

### Self-reflection

Naturalistic research may not always be experimentally tested and since the analysis is subjective, it may be influenced by researcher bias (Koch and Harrington, 1998; Ryan et al., 2007). By being reflexive, the qualitative researcher can confront and attempt to negate the effect of bias on the collection of data and final results (Flick, 2018).

The authors’ experiences in primary care have motivated their interest in this specific research question. DM acknowledges a strong emotional attachment to the programme and is aware of potential conflict of interest, firstly recognising the support from CAWT and NIAS enabling his educational transition to advanced paramedic over a three-year period. There is also DM’s determination to showcase this programme and the contribution paramedics can make to different areas of the health system. Nevertheless, the authors have endeavoured to construct a study that is auditable and transparent, with sufficient checks and balances to deter this turning into a self-derived project.

## Results and discussion

The thematic analysis of the interviews produced the following three superordinate themes: *alleviating pressure*;* acceptance*; and *psychological well-being*. Each will be defined and explored in turn by discussing the ordinate themes emerging form the data.

### Results

#### Superordinate theme of alleviating pressure

The superordinate theme of alleviating pressure is underpinned by themes of workload planning, collaboration and improving work–life balance.

The study participants felt that better workload planning would decrease their workload. The doctors recognised that this could be achieved by triaging patients, ensuring the patient gets to see or speak to the right clinician for their issue.

**Dr A:** . . . not all patients are suitable to be seen by the community paramedics but there is plenty that are . . . we could get the work done more efficiently . . .

The participants also commented on how the utilisation of Allied Health Professionals in primary care enabled better workload planning.

**Dr D:** . . . I believe that the involvement of advanced nurse practitioners or advanced or specialist paramedics are essentially the best hope there is for the future of the primary care model . . .

The GPs also made tangible links between being supported at work and reducing stress levels, resulting in a better work–life balance.

**Dr C:** . . . I have someone else to help me that day . . . And I think that’s actually a very important thing for everybody because we all know morale is very poor . . . that can make a day go from a horrible day to, that wasn’t such a bad day . . . you know I coped today rather than I was frazzled . . .

#### Superordinate theme of acceptance

The superordinate theme of acceptance is supported by themes of relationships and the fear of change.

The participants recognised the need to change how services were delivered to manage the demands of the current NHS primary care model. However, it was not known if this new paramedic initiative would be accepted by the patients or how it would complement existing GP services.

**Dr D:** A new role for paramedics in general practice is something we’ve been very fortunate to have the opportunity to be involved with here . . . I didn’t initially know how it would go until we were a little bit down the road . . .

Dr B believed if a patient was to be seen by the advanced paramedic the doctors in the practice needed to be sure the paramedic was suitably qualified for this new advanced role.

**Dr B:** . . . as a GP you are very protective of your skills and the amount of training you have had, but if you are going to have someone that is substituting you, you need to know . . . they have had good training.

The doctors in the study placed great value on the relationships with their patients, as these connections are often built up over many years. It was important to them that the paramedic developed similar links and gained the patients’ trust.

**Dr C:** . . . The patients enjoy the paramedics coming out because the paramedics certainly have more time to spend with them than the doctors have; the patients appreciate that and a lot of the time the paramedic can sit down and explain things to them . . . and that reassures them whereas . . . We don’t have that time . . . I’ve had a lot of positive feedback from patients saying that they like the interaction and they feel supported and feel reassured. They feel this has made a positive impact upon their care; I certainly think that we have kept people out of hospital . . .

#### Superordinate theme of psychological well-being

The superordinate theme of psychological well-being is supported by themes of mentorship and resilience.

Being able to control unexpected events at work was seen by participants as a way of building resilience. They felt this could be achieved by having an advanced paramedic on site.

**Dr C:** . . . and it’s a security blanket, you know that there is someone else here today to help . . . you feel as if your day is going to go better . . . I feel that I can deal more effectively with the workload . . .

Mentorship was reported by the participants as a way to facilitate increased collaboration between professions. The doctors readily admitted to getting emotional and psychological benefits from these interactions; firstly as a strategy to reduce stress, and secondly to enjoy the cognitive benefit of ‘keeping sharp’ (Dr B).

**Dr A:** I love the mentoring role and I thoroughly enjoy it, and I see that as a positive thing about being here, having you guys here . . .

And I enjoy that discussion, that educational opportunity between yourself and myself. It is a bit of a breath of fresh air . . .

### Discussion

The broadly positive experiences relayed by the doctors in the study appear to stem from the level of collaboration between the GPs and paramedics. Previous studies have consistently found that creating the right environment for collaborative practice to flourish needs careful management (Hillege et al., 2005). Morgan et al. (2015) also found that collaborative practice thrives in respectful environments, with the additional benefit of improving healthcare provision. Dr B reflected on how the successful collaboration between the doctors and paramedics was underpinned by the creation of a non-hierarchical relationship between the two professions. This philosophy is becoming more widespread and is in keeping with contemporary teamworking in the NHS (Jaruseviciene et al., 2013). The willingness of the participants to engage and work with the advanced paramedics has undoubtedly resulted in the success of the pilot programme. Many previous studies have demonstrated that the failure to engage with advanced practitioners by doctors has curtailed many programmes (Bryant-Lukosius et al., 2004; Hillege et al., 2005).

The perception of attaining a good work–life balance is linked to the feeling of being supported at work. A good work–life balance was reported to better prepare GPs for dealing with stress (Cheshire et al., 2017; Doran et al., 2016). Fisher et al. (2017) reported that two-thirds of GPs described their workload as ‘unsustainable’ and called for an increase in strategies to manage workload.

One strategy reported in previous studies to help the GP achieve a better work–life balance is delegating or task-shifting (Cheshire et al., 2017; Fisher et al., 2017; Karimi-Shahanjarini et al., 2019). Participants in the study reported that when they decreased their workload by delegating tasks to the advanced paramedic, they were happier, experienced improved job satisfaction and felt more supported. This approach echoes the vision set out in the 2015 Primary Care Workforce Commission report, which advocated for an increase in the multidisciplinary workforce to secure the future of primary care. The increased use of advanced paramedics in primary care has already been shown to be a sustainable method of delivering safe patient-centred care (Dainty et al., 2018).

Primary care is largely based around the management of chronic illness, which often results in a GP–patient dynamic that is built up over a lifetime of care. It is understandable that the GPs were anxious to stress the importance of relationship-building between the advanced paramedic and the patients. However, the study indicated that the GPs may have worried unnecessarily, as the feedback they received showed that the patients enjoyed the longer consultation times provided by the paramedics. Patients enjoying longer consultations in general practice is not a finding that is unique to this study; indeed, earlier studies have also reported similar results (Branson and Badger, 2008). The paramedics being accepted, and in some cases being asked for instead of a GP, correlates with previous studies reporting that patients were widely receptive of nurses substituting doctors for a variety of tasks (Ross, 2015). However, as Dr B relayed, the paramedic must be suitably trained for this expanded role. Indeed, nurses have previously deemed education and extra training as fundamental to expanding their roles and central to being accepted by other professions (Hillege et al., 2005). The importance of increased education as an enabler to advanced paramedic practice has also been relayed in previous studies (O’Meara et al., 2015).

The participants reported that being able to control unexpected events at work helped build resilience. Resilient doctors have been found to be more empathetic, to have better health, to less often entertain notions of quitting and to make fewer mistakes (Cheshire et al., 2017). The participants also reported feeling less stressed, less anxious, more secure, more optimistic and more empowered at work when they had an advanced paramedic as part of their primary care team.

## Strengths and limitations

This study provides an insight into the experiences of a small group of GPs from the same practice. However, their unique experiences may not necessarily be shared by other doctors in different primary care settings. Although the semi-structured interviews were designed to allow the participants sufficient latitude to cover in-depth their experiences of the programme, it was recognised that there was the potential for bias within the interview process. Firstly, the primary researcher (DM) conducting the interviews was one of the advanced paramedics involved in the pilot programme and worked with the interviewees. The responses given by the doctors may be biased as they may wish to see the programme being viewed as a success. Equally, the potential for author bias within the interview schedule and subsequent analysis is recognised. Therefore, to enhance rigour, clear auditable actions were embedded within the study design. The provision of a researcher (KS) to independently read the transcripts and agree on the thematic findings was introduced to reduce bias in reporting and strengthen the dependability of the study. Rigour may have been improved further by employing an independent interviewer; although, without a background in paramedicine and primary care work, an independent interviewer could be regarded as a limitation as their lack of direct experience could lead to misunderstandings and limited recognition of salient points within the interview discussion. The researcher endeavoured to create a relaxed and open environment in which participants felt comfortable enough to speak openly and honestly.

The small sample size may be viewed by some as a limitation; however, this qualitative descriptive study did not aim for generalisability. Instead, gaining a depth of understanding of the experience and its meaning to the participant was the key focus, which this small purposive sample allows.

## Conclusion

This qualitative descriptive study gives an insight into the complexity of general practice and the evolving nature of paramedicine within a multidisciplinary primary healthcare team. Generally, there was widespread support from the GPs for the introduction of advanced paramedics into primary care teams. The reasons were multi-factorial but the reduction in GP workload featured prominently. The participants reported benefits in terms of increased resilience and work–life balance. The capacity to provide a clinician with experience of dealing with acute and emergency presentations, in combination with managing routine procedures, was also reported to be of great importance. The study has not offered any clinical recommendations; however, it has indicated that paramedics have the abilities and skills to make a valuable contribution to primary care.

The study has highlighted opportunities to conduct future research examining the barriers and facilitators that GPs and advanced paramedics encounter when building collaborative practices. These topics could be examined using a combination of qualitative, quantitative and mixed methods research. Those engaged in research at undergraduate and postgraduate levels may find areas of interest, including examining if employing an advanced paramedic is a financially viable option in reversing the declining numbers of clinicians in primary care. Other topics that could be examined include whether advanced paramedics have similar rates of hospital admissions as their GP colleagues, or examining the views of advanced paramedics on rotational working. Research such as this will help to build a body of evidence that will assist key stakeholders including the Department of Health, Ambulance Services, Local Commissioning Groups and GP federations wishing to develop paramedicine and community healthcare provision. This could be in in primary care, out-of-hours services, urgent care centres or secondary care settings. The outcomes of future research will enable comparisons to be made with the findings of this initial study.

## Acknowledgements

A special thanks to Karen Seddon (KS) for her support and assistance with thematic analysis and proofreading. Also, thanks to Caroline French for her assistance during the interview process. This article is taken from DM’s MSc dissertation and represents a small portion of results recorded in a larger study.

## Author contributions

DM was the principal author of this study. CS was co-author and contributed to study conception, design, proofreading and manuscript editing. CS acts as the guarantor for this article.

## Conflict of interest

The principal author is involved in the pilot programme.

## Ethics

Ethical approval was gained from Glasgow Caledonian University (HLS/PSWAHS/19/027) and the Northern Ireland Ambulance Service to conduct the study. Ethical approval from the NHS Research Ethics Committee was not required as no patients were involved.

## Funding

The authors’ MSc studies were funded by CAWT. CAWT is a cross-border health and social care partnership for the Health Service Executive in the Republic of Ireland and the Southern and Western Health and Social Care Trusts, the Health and Social Care Board and the Public Health Agency in Northern Ireland.

## References

[bibr_1] Biggerstaff D. & Thompson A. R. (2008). Interpretative phenomenological analysis (IPA): A qualitative methodology of choice in healthcare research. *Qualitative Research in Psychology*, 5(3), 214–224.

[bibr_2] Booker M. & Voss S. (2019). Models of paramedic involvement in general practice. *British Journal of General Practice*, 69(687), 477–478. 10.3399/bjgp19X705605 PMC6774706 31558511

[bibr_3] Branson C. & Badger B. (2008). Skill mix in general practice: Christine Branson and Beryl Badger report on a four-year study. *Primary Health Care*, 18(1), 35–39.

[bibr_4] Braun V. & Clarke V. (2006). Using thematic analysis in psychology. *Qualitative Research in Psychology*, 3(2), 77–101.

[bibr_5] Brown P. (2017). A day in the life of a paramedic advanced clinical practitioner in primary care. *Journal of Paramedic Practice*, 9(9), 378–386.

[bibr_6] Bryant-Lukosius D. Dicenso A. Browne G. & Pinelli J. (2004). Advanced practice nursing roles: Development, implementation, and evaluation. *Journal of Advanced Nursing*, 48(5), 519–529. 15533090 10.1111/j.1365-2648.2004.03234.x

[bibr_7] Cheshire A. Ridge D. Hughes J. Peters D. Panagioti M. Simon C. & Lewith G. (2017). Influences on GP coping and resilience: A qualitative study in primary care. *The British Journal of General Practice: The Journal of the Royal College of General Practitioners*, 67(659), e428–e436. https://doi.org/10.3399/bjgp17X690893. 28483822 10.3399/bjgp17X690893 PMC5442958

[bibr_8] Dainty K. N. Seaton M. B. Drennan I. R. & Morrison L. J. (2018). Home visit-based community paramedicine and its potential role in improving patient centred primary care: A grounded theory study and framework. *Health Services Research*, 53(5), 3455–3470. 29542111 10.1111/1475-6773.12855 PMC6153157

[bibr_9] DeSantis L. & Ugarriza D. N. (2000). The concept of theme as used in qualitative nursing research. *Western Journal of Nursing Research*, 22(3), 351–372. 10804897 10.1177/019394590002200308

[bibr_10] Doran N. Fox F. Rodham K. Taylor G. & Harris M. (2016). Lost to the NHS: A mixed methods study of why GPs leave practice early in England. *The British Journal of General Practice: The Journal of the Royal College of General Practitioners*, 66(643), e128–e135. https://doi.org/10.3399/bjgp16X683425. 26740606 10.3399/bjgp16X683425 PMC4723211

[bibr_11] Fisher R. F. Croxson C. H. Ashdown H. F. & Hobbs F. R. (2017). GP views on strategies to cope with increasing workload: A qualitative interview study. *The British Journal of General Practice: The Journal of the Royal College of General Practitioners*, 67(655), e148–e156. https://doi.org/10.3399/bjgp17X688861. 28093421 10.3399/bjgp17X688861 PMC5308121

[bibr_12] Flick U. (2018). *An introduction to qualitative research*. Sage Publications Limited.

[bibr_13] Hillege S. Coulon L. Swann W. & Wilson K. (2005). Nurse practitioners’ experiences of working collaboratively with general practitioners and allied health professionals in New South Wales, Australia. *Australian Journal of Advanced Nursing*, 23(2), 22–27. 16502965

[bibr_14] Jaruseviciene L. Liseckiene I. Valius L. Kontrimiene A. Jarusevicius G. & Lapão L. V. (2013). Teamwork in primary care: Perspectives of general practitioners and community nurses in Lithuania. *BMC Family Practice*, 14(1), 118. 23945286 10.1186/1471-2296-14-118 PMC3751467

[bibr_15] Karimi-Shahanjarini A. Shakibazadeh E. Rashidian A. Hajimiri K. Glenton C. Noyes J. & Colvin C. J. (2019). Barriers and facilitators to the implementation of doctor-nurse substitution strategies in primary care: A qualitative evidence synthesis. *Cochrane Database of Systematic Reviews*, 4, CD010412. https://doi.org/10.1002/14651858.CD010412.pub2. 30982950 10.1002/14651858.CD010412.pub2 PMC6462850

[bibr_16] Koch T. (2006). Establishing rigour in qualitative research: The decision trail. *Journal of Advanced Nursing*, 53(1), 91–100. 16422698 10.1111/j.1365-2648.2006.03681.x

[bibr_17] Koch T. & Harrington A. (1998). Reconceptualizing rigour: The case for reflexivity. *Journal of Advanced Nursing*, 28(4), 882–890. 9829678 10.1046/j.1365-2648.1998.00725.x

[bibr_18] Larkin M. Watts S. & Clifton E. (2006). Giving voice and making sense in interpretative phenomenological analysis. *Qualitative Research in Psychology*, 3(2), 102–120.

[bibr_19] Martin A. C. & O’Meara P. (2019). Perspectives from the frontline of two north American community paramedicine programs: An observational, ethnographic study. *Rural & Remote Health*, 19(1), 1–9. 10.22605/RRH4888 30704256

[bibr_20] Morgan S. Pullon S. & McKinlay E. (2015). Observation of interprofessional collaborative practice in primary care teams: An integrative literature review. *International Journal of Nursing Studies*, 52(7), 1217–1230. 25862411 10.1016/j.ijnurstu.2015.03.008

[bibr_21] NHS England. (2014). *Five year forward view*. https://www.england.nhs.uk/wp-content/uploads/2014/10/5yfv-web.pdf.

[bibr_22] O’Meara P. Stirling C. Ruest M. & Martin A. (2015). Community paramedicine model of care: An observational, ethnographic case study. *BMC Health Services Research*, 16(1), 39. 10.1186/s12913-016-1282-0 PMC4739332 26842850

[bibr_23] Pillin H. (2015). The NHS oil tanker: Heading towards a new horizon for urgent and emergency care. *Journal of Paramedic Practice*, 7(2), 60–64.

[bibr_24] Ross J. (2015). Mental health nurse prescribing: The emerging impact. *Journal of Psychiatric and Mental Health Nursing*, 22(7), 529–542. 26031457 10.1111/jpm.12207

[bibr_25] Ryan F. Coughlan M. & Cronin P. (2007). Step-by-step guide to critiquing research. Part 2: Qualitative research. *British Journal of Nursing*, 16(12), 738–744. 17851363 10.12968/bjon.2007.16.12.23726

[bibr_26] Sandelowski M. (2000). Whatever happened to qualitative description? *Research in Nursing & Health*, 23(4), 334–340. 10940958 10.1002/1098-240x(200008)23:4<334::aid-nur9>3.0.co;2-g

[bibr_27] Sandelowski M. (2010). What’s in a name? Qualitative description revisited. *Research in Nursing & Health*, 33(1), 77–84. 20014004 10.1002/nur.20362

[bibr_28] Schofield B. Voss S. Proctor A. Benger J. Coates D. Kirby K. Purdy S. & Booker M. (2020). Exploring how paramedics are deployed in general practice and the perceived benefits and drawbacks: A mixed methods scoping study. *BJGP Open*, 4(2). https://doi.org/bjgpopen20X101037. 10.3399/bjgpopen20X101037 PMC7330225 32398344

[bibr_29] Smith J. A. (1996). Beyond the divide between cognition and discourse: Using interpretative phenomenological analysis in health psychology. *Psychology and Health*, 11(2), 261–271.

[bibr_30] Smith J. A. (2007). Hermeneutics, human sciences and health: Linking theory and practice. *International Journal of Qualitative Studies on Health and Well-being*, 2(1), 3–11.

[bibr_31] Smith J. A. Flowers P. & Larkin M. (2009). *Interpretative phenomenological analysis: Theory, method, and research*. Sage Publications.

